# An Investigation into the Rehabilitative Mechanism of Tuina in the Treatment of Sciatic Nerve Injury

**DOI:** 10.1155/2020/5859298

**Published:** 2020-07-17

**Authors:** Taotao Lv, Yanjun Mo, Tianyuan Yu, Yumo Zhang, Shuai Shao, Yuting Luo, Yi Shen, Mengqian Lu, Steven Gregory Wong

**Affiliations:** School of Acupuncture, Moxibustion and Tuina, Beijing University of Chinese Medicine, Beijing, China

## Abstract

**Objective:**

To explore the effect of tuina on the gene expression at the point of nerve injury in rats with sciatic nerve injury (SNI) and to elucidate the repair mechanism of tuina promoting the functional recovery of peripheral nerve injury.

**Methods:**

In the Sham group, the right sciatic nerve was exposed without clamping. The SNI model was established using the sciatic nerve clamp method on the right leg and then randomly divided into the SNI group and the Tuina group. Seven days after modeling, the Tuina group was treated daily with a “massage and tuina manipulation simulator” (Patent No. ZL 2007 0187403.1), which was used daily to stimulate Yinmen (BL37), Yanglingquan (GB34), and Chengshan (BL57) with point-pressing method, plucking method, and kneading method. The stimulating force was 4N, and the stimulating frequency was 60 times per minute; each method and each point were used for 1 minute, totaling 9 minutes (1 min/acupoint/method × 3 methods × 3 acupoints). Treatment was administered for 21 days, followed by a 1-day rest after the 10th treatment, for a total of 20 times of intervention. The sciatic function index (SFI) was used to evaluate the fine movements of the hind limbs of rats in each group. The ultrastructural changes at the point of nerve injury were observed by transmission electron microscopy, and the gene changes at the point of nerve injury were detected using RNA-sequencing (RNA-seq) technology.

**Results:**

Compared with the baseline, the SFI of the SNI group and the Tuina group decreased significantly at the 0th intervention (7 days after molding); compared with the SNI group, the SFI of the Tuina group increased at the 10th intervention (*P* < 0.05) and increased significantly at the 15th and 20th intervention (*P* < 0.01). Compared with the Sham group, the myelin sheath integrity of the sciatic nerve in the SNI group was destroyed and the myelin sheath collapsed seriously, even forming myelin sheath ball, accompanied with severe axonal atrophy and mitochondrial degeneration. The tuina intervention could significantly improve the ultrastructure of the nerve injury point, and the nerve fiber myelin sheath in the Tuina group remained intact, without obvious axonal swelling or atrophy. Atrophic thread granules could be seen in the axon, but there were no vacuolated mitochondria. RNA-seq results showed that there were differences at 221 genes at the point of nerve injury between the Tuina group and the SNI group and the differentially expressed genes (DEGs) are enriched in the biological processes related to the regulation of myocyte. Regulations include the regulation of striated muscle cell differentiation, myoblast differentiation, and myotube differentiation.

**Conclusion:**

Tuina can improve the fine motor recovery and protect the myelin integrity in rats with peripheral nerve injury, and this is achieved by changing the gene sequence at the injured point.

## 1. Introduction

SNI refers to the injury of the sciatic nerve or its branches caused by an external force, which is a common peripheral nerve injury (PNI). Clamp injury of the sciatic nerve is a classical model of sciatic nerve injury and a regeneration model of specific PNI [1–3]; it also can simulate II-III degree injury of peripheral nerve [4]. The axon and myelin sheath will break down and the nerve microfilament, microtubule, and axon outline will appear obviously irregular and disordered after the nerve injury [5]. These processes involve many kinds of cells and signal pathways, as well as a large number of gene expression changes [6]. In the early stage of injury, proliferating mast cells and Schwann cells clear the damaged axon and deformed myelin sheath by releasing cytokines such as interferon-*γ* (IFN-*γ*), promote the aggregation of macrophages, and speed up the degeneration process of the distal end of the nerve fibers [7]. Damaged peripheral nerves are renewable [8], and under the normal conditions of the structure and function of the nerve cell body, the regenerated axon can be induced to extend through the damaged area. The axon growth cone can grow into the effector to find and identify the target organ, reconstruct the complete axon, and promote its full regeneration and functional recovery [9, 10]. In general, nerve function can only recover about 70% after microsurgical repair, cell transplantation, and the use of nerve growth factor. Tuina is a kind of external therapy belonging to traditional Chinese medicine, in which the therapist issues a mechanical force with the appropriate amount of stimulation on the surface or directed to the deep tissue of the subject. This stimulation causes changes to the receptor, which then transforms the mechanical stimulation into electrical signals, reaching the central nervous system through the afferent fiber in the form of nerve impulses. Cells of the immune system are affected by forces and pressures throughout their life cycle, but how these mechanical processes regulate the immune response has not been clearly revealed [11]. The combination of tuina and treadmill training can change the conduction velocity of the sciatic nerve and the number of regenerated axons and Schwann cells [12]. Tuina can promote the regeneration of the myelin sheath and axons of the sciatic nerve fiber in SNI rats, alleviate the edema of Schwann cell cytoplasm and mitochondria, and promote the repair and regeneration of the injured nerve ultrastructure [13]. Tuina can improve the behavioral indexes of SNI rats, promote the expression of neurotrophic factors (NTFS), protect neurons, prevent muscle atrophy, and significantly promote the recovery of SNI [14, 15]. RNA-sequencing (RNA-seq) is a technique for sequence detection from transcriptional information formed into the transcription process of specific tissues and cell types. It can compare the transcriptome in different stages or parts from biological samples, so as to reflect changes in the level of gene expression of different genes at the transcription level. RNA-seq can study gene function and gene structure at the overall level and reveal the molecular mechanism of specific biological and pathological processes.

The sciatic nerve clamped injury model was used to simulate clinical SNI in this study. Seven days after the establishment of the model, point-pressing, plucking, and kneading methods were used to stimulate Yinmen (BL37), Chengshan (BL57) and Yanglingquan (GB34) points on the affected side of the rats. SFI was used to explore the recovery of fine movement of the hind limbs of the SNI rats, ultrastructural changes at the point of injury were observed using electric microscopy, and RNA-seq technology was used to study the transcription changes at the point of injury while the tuina intervention was applied, to explain the mechanism of tuina in promoting SNI recovery and provide a novel approach for the treatment of PNI.

## 2. Methods

### 2.1. Animals and Modeling Methods

Forty-five male Sprague Dawley (SD) rats weighing 200 ± 10 g were obtained from SBF Biotechnology Co., Ltd., and were raised in the laboratory of Beijing University of Chinese Medicine (BUCM), where food and water were freely provided. The temperature was stabilized at 23 ± 2°C, the humidity was 45%, and the rats were raised in 12°h light/12°h dark. Adaptive feeding was administered for 1 week. The SD rats used in the study were divided into three groups with 15 rats in each group: sham operation group (Sham group), model group (SNI group), and treatment group (Tuina group). Six rats in each group were used for electron microscopy to observe the structure at the point of injured nerve, and nine fresh tissues in each group were used for sequencing analysis. The animals were treated humanely and with regard to alleviation of suffering, and the study was approved by the Committee on Animal Protection and Use of BUCM (BUCM-4-2018101902-4010).

Rats were subjected to fasting and water prohibition for 24°h before the surgery [15] and were anesthetized by intraperitoneal injection of 1% pentobarbital sodium (0.35 mL/100 g).

Rats of Sham group were fixed in a prone position, and the skin at the right hip femoral junction was prepared and disinfected with Iodophor. A skin incision of about 1 cm along the direction of the sciatic nerve was conducted, and blunt separation of the muscle layer was performed, which fully exposed the lower edge of the piriformis muscle. At 5 mm distal of the sciatic nerve node, the sciatic nerve was exposed [16]; layer by layer was disinfected, sutured, and then disinfected.

The sciatic nerves of rats of SNI group and Tuina group were found in the same way as the Sham group and then clamped for 5 s with special fine toothless forceps, with full strength (6 kg through pressure test), resulting in a 2°mm wide injury point. We performed local normal saline wash and layer by layer disinfection; then, the skin was sutured with 4–0 stitches [14], and redisinfection was conducted; the rats were kept warm and we waited until they regained consciousness.

### 2.2. Intervention Method

The SNI group and Sham group were fed routinely and they were held in the restraint for 9 minutes, but with no tuina intervention.

The Tuina group began treatment on the 7th day after the operation. Three tuina methods with qualitative and quantitative controls were administered daily: point-pressing, plucking, and kneading methods were administered using a “Massage and Tuina manipulation simulator,” which has smooth spherical surface with a diameter of 10 mm to stimulate Yinmen (BL37), Chengshan (BL57), and Yanglingquan (GB34) points on the affected side of the rats; the stimulating force was 4°N and the stimulating frequency was 60 times per minute [17]. Each method and each point were used for 1 minute, totaling 9 minutes (1 min/acupoint/method × 3 methods × 3 acupoints). Treatment was administered once a day for 10 days, with 1 day of rest, and then 10 more treatments for a total of 20 times. In order to reduce the stress response of animals, petting and catching of the animals were administered for 9 minutes every day before the formal intervention.

### 2.3. Behavioral Measures

SFI was measured at baseline, before the 0th, and after the 5th, 10th, 15th, and 20th interventions, respectively, to evaluate the recovery of fine motor ability of the hind limbs of rats in each group. The specific assessment method was as follows: immerse the hind limbs of rats in pigment, put the rats in a white paper trough, and make them walk to the opposite side of the trough by themselves, leaving 5-6 footprints on each side. The footprints of the experimental side foot (*E*) and the normal side foot (*N*) were selected to measure three variables: the print length (PL), the toe spread (TS), and the intermediary toe spread (IT). Using the Bain formula, SFI = 109.5 (ETS-NTS)/NTS-38.3 (EPL-NPL)/NPL + 13.3 (EIT-NIT)/NIT-8.8. Each set of data is measured to the nearest millimeter, which is calculated by the Bain formula. SFI = 0 is the normal value and SFI = −100 represents complete nerve disconnection.

### 2.4. Tissue Sample Preparation and Electron Microscopy

After 20 times of intervention, 6 rats were taken from each group. Rats were anesthetized by intraperitoneal injection of 1% pentobarbital sodium (0.35 ml/100 g). Blood was collected from the abdominal aorta and the rats were quickly placed on ice. The point of injury on the right sciatic nerve was taken and fixed in 3% glutaraldehyde for 2 hours before rinsing and then fixed in 1% osmium acid for 1.5 hours. It was stained with uranyl acetate for 1 h. Dehydration with 50%–100% alcohol was done step by step for 15 mins. Pure alcohol : pure acetone (1 : 1) and pure acetone dehydration were performed for 10 min in each step. Samples were then embedded with epoxy resin and sliced with a Leica ultrathin microtome (50–70 nm). After lead staining, a JEM-100 electron microscope was used to observe the ultrastructural changes at the point of nerve injury, and a CCD digital photography system was used to collect images.

### 2.5. RNA Extraction and Construction of Sequencing Library

Total RNA was extracted using the standard TRIzol extraction procedures. The nerves in every three rats were collected as one sample to extract the total RNA, and each group had three biological repeats [18]. NanoDrop 2000 was used to detect the concentration and purity of RNA. Agarose gel electrophoresis was used to assess the integrity of the RNA. Agilent 2100 was used to determine the integrity of the RNA (RIN). The total amount of RNA required for a single database construction is 1 ug; the concentration ≥50 ng/*μ*L, OD260/280 between 1.8 and 2.2, and the score of RIN >8 were used for sequencing. In this project, transcriptome sequencing was completed based on the HiSeq sequencing platform, and the Illuminape library was constructed by the Illumina TruSeqTM RNA sample prep kit method for 2 × 150 bp sequencing. Quantizing was conducted with QuantiFluor® dsDNA System and combining with the computer according to the data proportion; bridge PCR amplification was conducted on CBot to generate clusters. After quality control analysis, bioinformatics was used to analyze the transcriptome data. Fragments per kilobase of exon model per million mapped reads (FPKM) value was used to measure gene expression level, and differential gene expression analysis was carried out on the whole transcriptome data of each sample.

### 2.6. Statistical Analysis

SPSS 22.0 was used to analyze the SFI values, and the results were represented by mean ± standard deviation ($\bar{x}\pm s$). Data were done by one-way ANOVA because data showed normal distribution. A difference was statistically significant with *P* < 0.05 and very significant with *P* < 0.01.

Goatools software was used to analyze the gene ontology (GO) enrichment of genes in the gene set. Fisher's accuracy test was used. The significantly enriched GO function was when the corrected *P*-adjust < 0.05. The *R* script was used to analyze the enrichment of genes in the Kyoto Encyclopedia of Genes and Genomes (KEGG); the calculation principle was the same as that of GO function enrichment analysis.

## 3. Results

### 3.1. Behavioral Results

**Figure 1:**
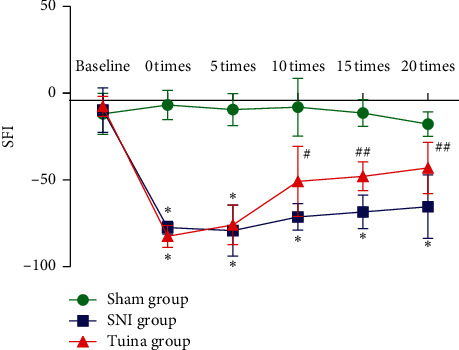
SFI results of rats in each group. Comparison with the SNI group: ^#^*P* < 0.05; ^##^*P* < 0.01. The abscissa is the number of interventions and the ordinate is the SFI value.

There was no redness or swelling point of injury for rats in each group, and the health status was good after the modeling operation. Compared with the baseline, the SFI of the SNI group and the Tuina group decreased significantly before intervention. After the SNI model was established, the sciatic nerve injury of the rats tended to be about 80%, and the fine motor function was greatly affected, indicating that the model was successfully prepared. Make the baseline before molding. The SFI results of rats in each group showed that, compared with the baseline, the SFI of rats in the SNI group and the Tuina group decreased significantly before intervention (7 days after operation); compared with the SNI group in the 10th intervention, the SFI of rats in the Tuina group increased significantly (*P* < 0.05) and the SFI of rats in the Tuina group increased very significantly (*P* < 0.01) in the 15th and 20th intervention (Figure 1). The results showed that tuina intervention can effectively improve the coordination function of the muscles in the lower limbs after nerve injury. Tuina can promote the recovery of fine movements in the hind limbs of rats and facilitate the recovery of motor function of nerve injury.

### 3.2. Ultrastructural Changes at the Point of Nerve Injury Observed via an Electron Microscope

**Figure 2:**
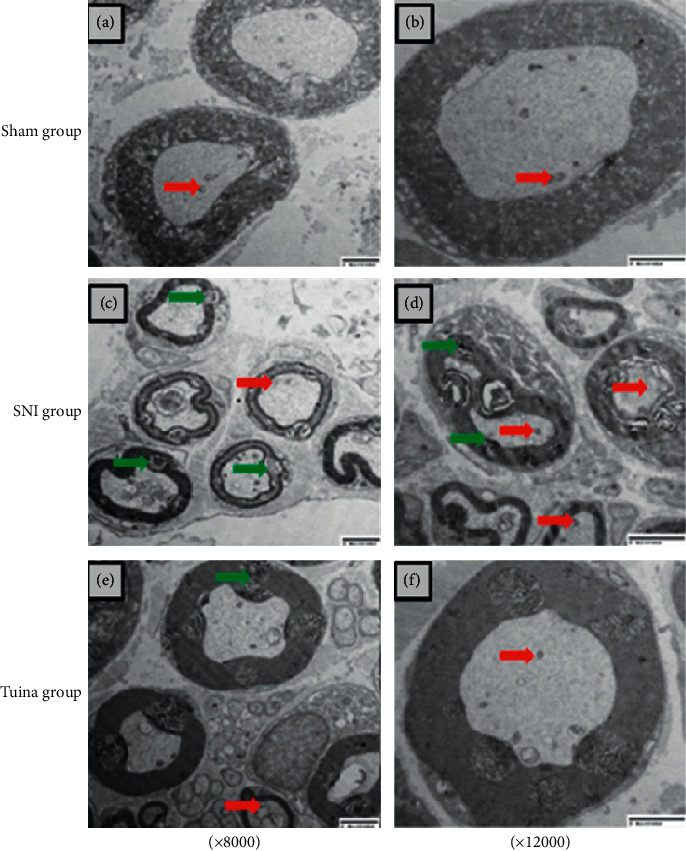
Electron microscopic observation of sciatic nerve injury points in each group. (a, b) Sham group. (c, d) SNI group. (e, f) Tuina group. 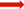
Autophagic body. 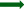
Myelin sphere.

The Sham group (Figures 2(a) and 2(b)): under the electron microscope, the structure of sciatic nerve is complete; the myelin sheath of nerve fiber is dense, evenly distributed, regular in shape, and lamellar in structure; the myelin sheath is thick and arranged orderly; and the axon is not atrophic or swollen.

The SNI group (Figures 2(c) and 2(d)): under the electron microscope, most of the myelin sheaths of the axons showed severe myelin collapse, twisted myelin sheaths, whirlpools, or even myelin balling or vacuole degeneration. The cytoskeleton was flocculent; axons were atrophied or even disappeared; mitochondria in axons were vacuolated; lamellar space was expanded and showed lamellar separation; the cytoplasm of Schwann cells was obviously dissolved; and autophagy and phagosomes were occasionally seen.

The Tuina group (Figures 2(e) and 2(f)): under the electron microscope, most of the myelin sheathes of nerve fibers were intact, part from the myelin sheath phospholipids fell off, occasionally the myelin sheath collapsed, and the inside of the myelin sheath was edematous. The axon was slightly atrophic and the atrophic mitochondria were visible, the structure was not clear, but there was no vacuolated mitochondria. The Schwann cell mitochondria were slightly swollen and the inside structure was visible. About half of the axon showed the formation of autophages.

### 3.3. Results of Bioinformatics Analysis at the Point of Nerve Injury in Rats

**Figure 3:**
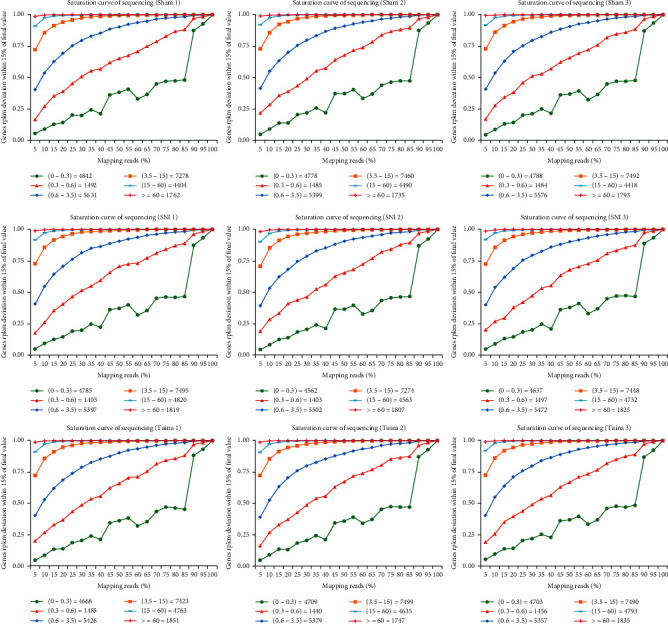
Saturation curve of each sample. The abscissa is the percentage of effective comparison reads. The vertical axis is the deviation ratio between the expression and the final value (within 15%); the closer the value is to 1, the more saturated the expression is. Each color line represents the saturation curve of gene expression at different expression levels in the sample. The genes with medium or above expression level, i.e., the genes with FPKM value above 3.5, are close to saturation (the vertical axis value approaches 1) when compared with 40% of sequenced reads, indicating that the overall quality of saturation is high, and the sequencing level can cover most of the expressed genes.

High throughput sequence technology was used to evaluate the quality of gene expression of each sample. A total of 280 million reads were obtained, with an average of 47.26 million reads per sample. Q20 of all samples ranged from 97.33% to 98.27%, and Q30 ranged from 92.25% to 94.63%. 96.65% to 97.07% of each sample could map to the reference genome, indicating that the sequence results were reliable (Table 1). We created saturation curves for each of our sequencing files to assess whether we have sufficient sequencing depth for downstream analysis (Figure 3).

**Figure 4:**
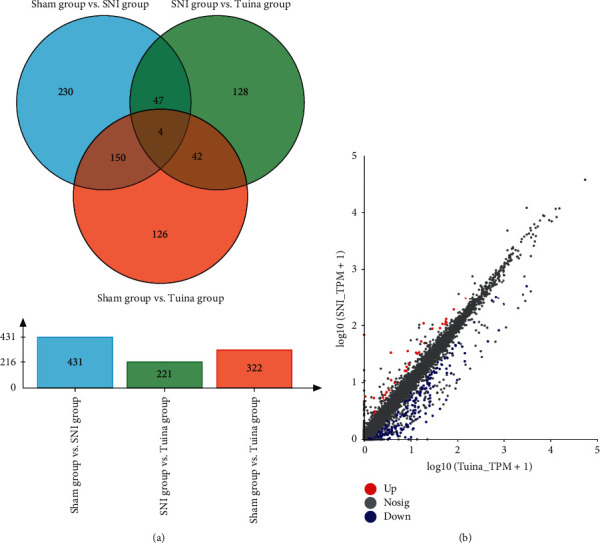
Veen analysis and Scatter diagram of expression difference. (a) Veen analysis: different gene sets are represented by circles of different colors, the values represent the number of genes in common among gene sets, and the cross areas represent the number of genes in common among gene sets. (b) Scatter diagram of expression difference between Tuina group and SNI group: the horizontal and vertical coordinates represent the gene expression amount in the sample, and each point represents a specific gene. The abscissa value of a point is the expression amount of the gene in the pair.

**Figure 5:**
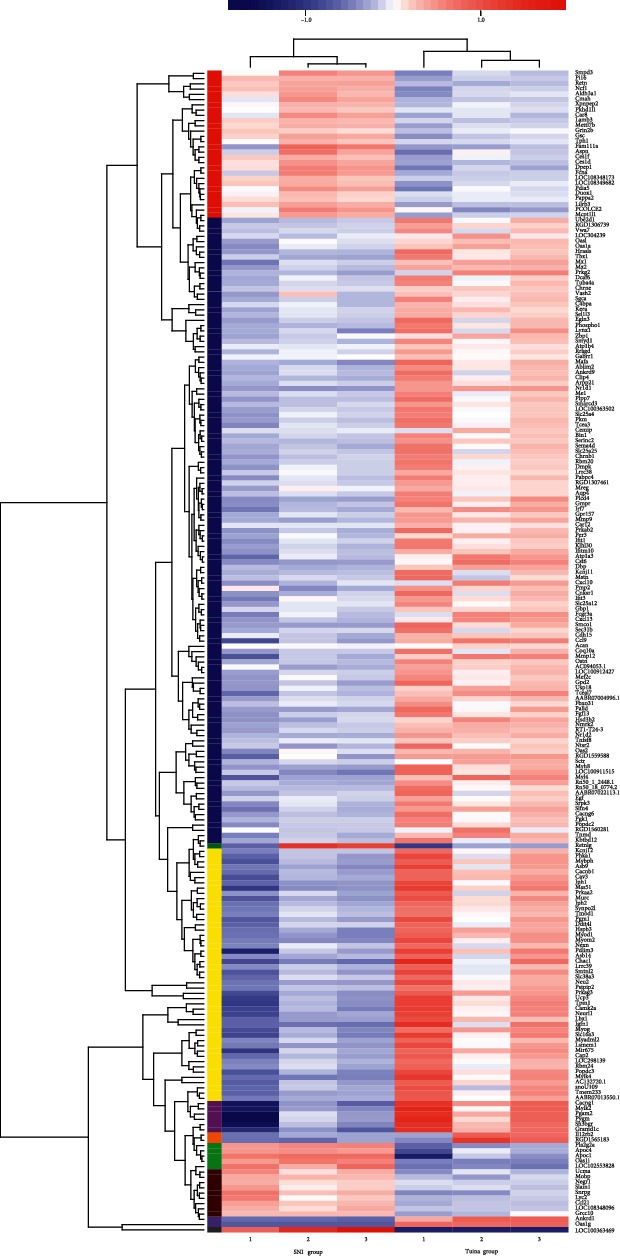
Two-dimensional hierarchical clustering analysis chart. Each column represents a sample, each row represents a gene, and the color in the figure represents the amount of gene expression in the sample (log_10_ (TPM + 1)): red represents the high amount of gene expression in the sample and green represents the low amount of expression. See the color bar above for the specific change trend of expression amount.

After 20 times of intervention, RNA sequence analysis was carried out on the genome at the point of nerve injury of both groups of rats (*P* value adjusted <0.05; up/down variance multiple is 2). Veen analysis results show that (Figure 4(a)) a total of 431 genes in the Sham group have significant difference changes compared with the SNI group; 221 genes in the SNI group have significant difference changes compared with the Tuina group; 322 genes in the Sham group have significant difference changes compared with the Tuina group. In the SNI group and Tuina group, 44 differential expression genes were upregulated and 177 differential expression genes were downregulated (Figure 4(b)). Two-dimensional hierarchical cluster analysis can clearly show the degree of separation between the SNI group and the Tuina group (Figure 5).

**Figure 6:**
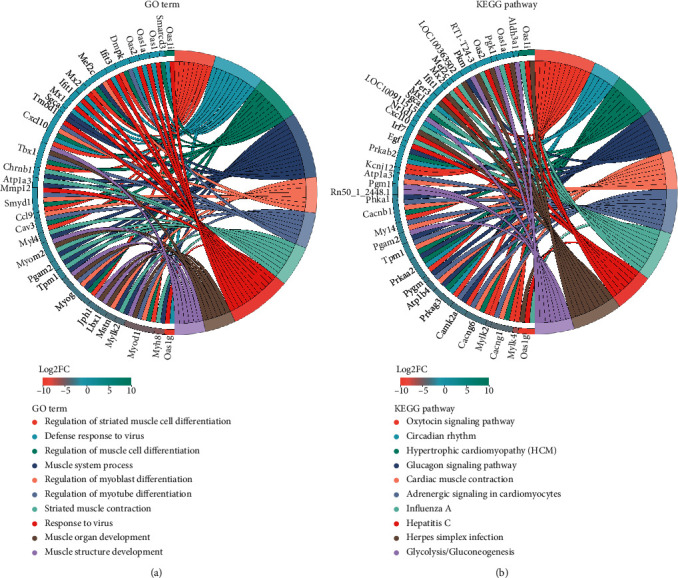
GO enrichment chord and KEGG enrichment chord. (a) The differentially expressed genes correspond to the significantly enriched GO terms. The left side is the genes, which are arranged in the order of log2FC from large to small. The larger log2FC is, the greater the differentially expressed multiple of the upregulated genes is, and the closer log2FC is to 0, the smaller the differentially expressed multiple of the genes is. The right side is the GO term on the significantly enriched differential genes information. (b) The differentially expressed genes correspond to the significantly enriched pathway, the left side is the same as GO term, and the right side is the KEGG pathway information on the significantly enriched DEGs.

Compared with the SNI group and Tuina group, the functions of GO are enriched in the regulation of striated muscle cell differentiation, defense response to viruses, regulation of muscle cell differentiation, muscle system processes, regulation of myoblast differentiation, regulation of myotube differentiation, striated muscle contraction, muscle organ development, and muscle structure development (Figure 6(a)).The largest differences between upregulated and downregulated expression were Oas1i(2′-5′oligoadenylate synthetase 1I) and Oas1g(2′-5′oligoadenylate synthetase 1G). The genes most involved in biological function are Myog (myogenin) and Myod1 (myogenic differentiation 1). KEGG pathway is enriched in oxytocin signaling pathway, circadian rhythm, hypertrophic cardiomyopathy (HCM), glucagon signaling pathway, cardiac muscle contraction, adrenergic signaling in cardiomyocytes, influenza A, hepatitis C, herpes simplex infection, and glycolysis (Figure 6(b)).

## 4. Discussion

PNI can cause nerve conduction problems, numbness of limbs, and the loss of autonomic control of corresponding body areas. As a kind of peripheral intervention, the effectiveness of tuina in the treatment of PNI has been established [19–21]. The mechanism of tuina in the treatment of PNI has not been clarified. In this study, the tuina techniques were selected and point-pressing method functions as a warming and general technique, while the plucking method and kneading method function as tissue relaxing techniques [22]. According to the course of the sciatic nerve, Yinmen (BL37), Chengshan (BL57), and Yanglingquan (GB34) points were selected as stimulation points. The three methods are helpful to relieve pain and muscle spasm at the injury site [23], and they can stimulate “acupoint-nerve-muscle” related areas at the same time (Table 2).

The results of SFI measurement showed that the coordination function of the hind limbs decreased after SNI, and the fine movement of the hind limbs was affected, so the SFI value decreased. The intervention of tuina can increase the SFI value. This study showed that tuina can promote the recovery of fine motor function of hind limbs in rats with SNI. The results of electron microscopy showed that the SNI model seriously affected the integrity of the myelin sheath of the sciatic nerve and the myelin sheath collapsed seriously, even forming myelin spheres, accompanied with severe axonal atrophy and mitochondrial degeneration. The ultrastructure of the points of injury in the Tuina group was significantly improved, the myelin sheath of the nerve fibers remained relatively intact, the axons were not obviously swollen or atrophic, and atrophic mitochondria were seen in the axons, but there was no vacuole line granulosis. It is suggested that tuina can promote the recovery of the ultrastructure at the point of injury of the sciatic nerve.

Understanding the transcriptional response of tissue to injury is helpful to understand the pathology of the disease. In this study, two-dimensional hierarchical cluster analysis can clearly show the degree of separation between the SNI group and the Tuina group, indicating that tuina can change the gene expression of neural damage point in SNI model rats. GO enrichment analysis confirmed that, under the action of tuina, the differentially expressed genes at the point of nerve injury were enriched in multiple biological processes, which indicated that tuina promoted the repair of peripheral nerve injury in various ways. It was found that the biological functions focused on the regulation of muscles such as regulation of striated muscle cell differentiation, regulations of muscle cell differentiation, muscle system process, regulation of myoblast differentiation, regulation of myotube differentiation, striated muscle contraction, and muscle organ development. Tuina can promote the repair of peripheral nerve injury, which may be related to the regulation of muscle cells. Skeletal muscle is dominated by the peripheral nerves, and its morphology, structure, and contraction function are regulated by motor nerves. After denervation of skeletal muscle, denaturation and dissolution of the nucleus, mitochondria, and various muscle-specific proteins, thinning of muscle fiber diameter and reduction of quantity, and fibrosis of skeletal muscle also occur. This is consistent with the result that the tuina can promote the recovery of denervated muscle. Previous research shows that there are obvious changes in cell diameter, intercellular factors, distribution, number of nuclei, atrophy degree of muscle cells, wet weight of muscle cells, and recovery rate of muscle cells in SNI rats after tuina intervention [24–26], as well as a decrease in the expression of CNTF, BFGF, and BDNF in the gastrocnemius [27]. Among the enrichment results of GO, the difference multiple of upregulated and downregulated expression was the largest in Oas1i (ENSRNOG00000037744) and Oas1g (ENSRNOG00000047076), whose biological functions were virus defense reaction and striated muscle contraction, and the KEGG pathway was enriched in influenza A, hepatitis C, and the virus defense reaction. Myog (ENSRNOG00000030743) and Myod1 (ENSRNOG00000011306) are the most important genes involved in biological function. They are involved in the regulation of striated muscle cell differentiation, regulation of muscle cell differentiation, regulation of myoblast differentiation, regulation of myotube differentiation, muscle organ development, and muscle structure development. Myog is also involved in the processes of the muscle cell system and the response of denervation to muscle adaptation regulation. KEGG was enriched in hypertrophic cardiomyopathy, the glucagon signaling pathway, cardiac muscle contraction, adrenergic signaling in cardiomyocytes, and other pathways related to the regulation of cardiomyocytes. Whether tuina intervention can regulate cardiac myocytes remains to be further studied. KEGG is also enriched in influenza A, hepatitis C, the viral defense response, and other pathways related to viral regulation. It is suggested that tuina can promote the recovery of PNI by regulating the local inflammatory changes of nerve injury point. This study elucidated the mechanism of tuina on the injured point of SNI and provided a theoretical basis for tuina to promote PNI repair and regeneration.

## 5. Conclusion

In this study, SD rats were used as experimental animals, a SNI model was used to simulate clinical peripheral nerve crush injury, tuina was used as the intervention mode, RNA-seq technology was used to explore the changes of gene sequence at the point of injury in rats with tuina intervention, and the molecular mechanism of tuina promoting PNI recovery was revealed. The study implies that tuina on SNI rats can improve the fine movement of hind limbs and the ultrastructure at the point of injury, change gene expression at the point of injury, and promote axon growth of the injured nerve, all leading to the improvement of the PNI, which can be achieved through a variety of GO functions and multiple KEGG channels. The results of this study support the view that RNA-seq analysis of differential gene expression provides the repair mechanism of PNI. In the future, we aim to further study the genes that showed significantly different expression and their related functions and improve the understanding of the mechanism of tuina in the repair of PNI and provide theoretical basis for the possible target of tuina in the treatment of PNI.

## Figures and Tables

**Table 1:** Quality control and sequencing information of each sample.

	Sample	Raw reads	Clean reads	Q20 (%)	Q30 (%)	Total mapped
1	Sham 1	45996402	45488638	98.24	94.53	44143048 (97.04%)
2	Sham 2	49336984	48784674	98.13	94.3	47295661 (96.95%)
3	Sham 3	48433120	47697864	97.67	93.18	46130890 (96.71%)
4	SNI 1	48311290	47689406	97.73	93.32	46140997 (96.75%)
5	SNI 2	45962802	45466708	98.27	94.63	44134471 (97.07%)
6	SNI 3	46637582	46017056	97.33	92.25	44506803 (96.72%)
7	Tuina 1	46364082	45634588	97.62	93.11	44106643 (96.65%)
8	Tuina 2	46912118	46294222	97.89	93.7	44757906 (96.68%)
9	Tuina 3	49393696	48764848	97.95	93.83	47199260 (96.79%)

Sample: 9 cDNA libraries are Sham group (Sham 1, 2, and 3), SNI group (SNI 1, 2, and 3), and Tuina group (Tuina 1, 2, and 3). Raw reads: amount of original sequence data. Clean reads: amount of sequenced data after filtering. Q20, Q30: percentage of bases greater than 20 and 30 in the total base. Total mapped: the number of clean reads that can be located on the genome.

**Table 2:** Acupoint nerve muscle related area.

Number	Acupoint	Nerve	Muscle
1	Yinmen (BL37)	Sciatic nerve	Biceps femoris
2	Chengshan (BL57)	Tibial nerve	Gastrocnemius
3	Yanglingquan (GB34)	Common peroneal nerve	Tibialis anterior

## Data Availability

The data used to support the findings of this study are available from the corresponding author upon request.
